# Mouse mammary tumors display Stat3 activation dependent on leukemia inhibitory factor signaling

**DOI:** 10.1186/bcr1777

**Published:** 2007-10-10

**Authors:** Ana Quaglino, Carolina Schere-Levy, Leonardo Romorini, Roberto P Meiss, Edith C Kordon

**Affiliations:** 1 IFIBYNE (CONICET), Facultad de Ciencias Exactas y Naturales, University of Buenos Aires, Argentina; 2 IIHEMA-IEO, Academia Nacional de Medicina, Buenos Aires, Argentina

## Abstract

**Introduction:**

It has been demonstrated that leukemia inhibitory factor (LIF) induces epithelium apoptosis through Stat3 activation during mouse mammary gland involution. In contrast, it has been shown that this transcription factor is commonly activated in breast cancer cells, although what causes this effect remains unknown. Here we have tested the hypothesis that locally produced LIF can be responsible for Stat3 activation in mouse mammary tumors.

**Methods:**

The studies were performed in different tumorigenic and non-tumorigenic mammary cells. The expression of LIF and LIF receptor was tested by RT-PCR analysis. In tumors, LIF and Stat3 proteins were analyzed by immunohistochemistry, whereas Stat3 and extracellular signal-regulated kinase (ERK)1/2 expression and phosphorylation were studied by Western blot analysis. A LIF-specific blocking antibody was used to determine whether this cytokine was responsible for Stat3 phosphorylation induced by conditioned medium. Specific pharmacological inhibitors (PD98059 and Stat3ip) that affect ERK1/2 and Stat3 activation were used to study their involvement in LIF-induced effects. To analyze cell survival, assays with crystal violet were performed.

**Results:**

High levels of LIF expression and activated Stat3 were found in mammary tumors growing *in vivo *and in their primary cultures. We found a single mouse mammary tumor cell line, LM3, that showed low levels of activated Stat3. Incidentally, these cells also showed very little expression of LIF receptor. This suggested that autocrine/paracrine LIF would be responsible for Stat3 activation in mouse mammary tumors. This hypothesis was confirmed by the ability of conditioned medium of mammary tumor primary cultures to induce Stat3 phosphorylation, activity that was prevented by pretreatment with LIF-blocking antibody. Besides, we found that LIF increased tumor cell viability. Interestingly, blocking Stat3 activation enhanced this effect in mammary tumor cells.

**Conclusion:**

LIF is overexpressed in mouse mammary tumors, where it acts as the main Stat3 activator. Interestingly, the positive LIF effect on tumor cell viability is not dependent on Stat3 activation, which inhibits tumor cell survival as it does in normal mammary epithelium.

## Introduction

The pleiotropic cytokine leukemia inhibitory factor (LIF) is a secreted 38 to 67 kDa glycoprotein first named for its ability to induce macrophage differentiation in the murine myeloid leukemic cell line M1 [1]. This factor has been detected in a variety of adult mouse tissues and displays different biological activities, including effects on bone metabolism, inflammation, neural development and embryogenesis [2]. A potential role for LIF in the pathogenesis of human breast cancer is indicated by its expression in breast cancer cells [3,4], which can be modulated by progestins and antiprogestins [5], and by its capacity to induce the proliferation of several estrogen-dependent (MCF-7 and T47D) and estrogen-independent (SK-BR3 and BT20) breast cancer cell lines as well as fresh breast carcinoma cells [4,6]. In spite of these data, little is known about the relevance of LIF for mammary tumor development *in vivo*.

Biological functions of LIF are mediated by the formation of a cell surface LIF receptor complex between the low-affinity LIF receptor (LIF-R) and a gp130 subunit [7,8]. All the known receptors that contain gp130 have Janus kinase (JAK) kinases (tyrosine kinases) bound to their intracellular tails [9]. After ligand-mediated receptor assembly, the JAKs become activated and phosphorylate cytoplasmic proteins called signal transducer and activators of transcription (Stats). The activated Stats then dimerize, translocate to the nucleus, and participate in transcriptional regulation by binding to specific DNA sites. It has been reported that among the seven members of the Stat family, Stat3 is the major mediator of gp130 signals [10,11].

In the normal mouse mammary gland, Stat3 is pro-apoptotic and a crucial mediator of post-lactational regression. Mammary local factors stimulate the phosphorylation of Stat3 during involution [12], and mammary glands of Stat3 conditional knockout mice showed a suppression of epithelial apoptosis that led to a marked delay in mammary gland involution [13]. However, elevated Stat3 tyrosine phosphorylation and DNA-binding activity have been reported in breast cancer cell lines. In addition, inhibition of the activation of Stat3 blocked the proliferation and survival of those cancer cells [14-16].

It has been established that LIF is the physiological activator of Stat3 during mammary gland involution and has a principal role in the apoptotic process [17,18]. In addition, the capacity of LIF to induce Stat3 phosphorylation has been demonstrated in several different experimental models [19-23]. However, no linkage has yet been made between LIF expression and Stat3 activation in mammary tumors. To address this issue, in the present study we evaluated LIF expression and its ability to induce Stat3 tyrosine phosphorylation in mouse mammary tumors. Taking into account the drastic difference in the significance of activation of this transcription factor in neoplastic and normal mammary cells, various tumor lines were assayed and compared with the non-tumorigenic HC11 cell line. We also studied LIF expression and its ability to induce Stat3 activation in mouse mammary tumor models with different grades of differentiation and malignancy: the non-metastatic mouse mammary tumor virus (MMTV)-induced hormone-dependent and hormone-independent neoplasias (HDTs and HITs, respectively) [24,25] and the poorly differentiated highly metastatic LM3 mammary tumor line [26].

## Materials and methods

### Animals

Female BALB/c mice from our mouse colony, 8 to 12 weeks in age and 20 to 25 g in weight, were used throughout. They were housed four per cage in conditioned rooms at 20 ± 2°C, kept under an automatic 12 hours light/12 hours darkness schedule, and given pellets and tap water *ad libitum*. All animal studies were conducted in accordance with the NIH Guide for the Care and the Use of Laboratory Animals.

### Tumors and cell lines

The tumors, primary cultures and cell lines used are described in Table 1.

**Table 1:** Tumors, primary cultures and cell lines used in the experiments in this paper

Source	Description	References
Tumors and primary cultures		
HDTs	MMTV(LA)-induced HDTs growing *in vivo*	[24,25,31]
HITs	MMTV(LA)-induced HITs growing *in vivo*	[24,25,31]
TPC	Primary cultures derived from HITs	
LM3 tumors	Poorly differentiated adenocarcinomas derived from LM3 cells implanted subcutaneously in BALB/c mice	
Cell lines		
LM3	Cell line established from a spontaneous mammary tumor in BALB/c mouse	[47]
LMM3	Cell line derived from a highly metastatic mammary tumor in BALB/c mouse	[47]
HC11	Normal mouse mammary epithelial cell line	[50,51]
SCP2	Normal mouse mammary epithelial cell line	[52]
NMuMG	Normal mouse mammary epithelial cell line	ATTC – CRL-1636™
MCF-7	Human ER^+ ^breast cancer cell line	ATTC – HTB-22™

HDT, hormone-dependent tumor; HIT, hormone-independent tumor; TPC, tumor primary culture; MMTV, mouse mammary tumor virus; ER, estrogen receptor, ATCC^®^, American Type Culture Collection Number.

Tumors growing *in vivo *were removed from mice before reaching 1 cm^3^, then cut into fragments and processed for the different experimental procedures.

### Cell culture assays

HC11 cells were maintained in growth medium (RPMI 1640 medium augmented with 10% fetal bovine serum (FBS; Invitrogen, Carlsbad, CA, USA), 5 μg/ml insulin (Sigma, Saint Louis, MO, USA) and 2 mM glutamine (Hyclone). MCF-7 and NMuMG cells were purchased from the American Type Culture Collection (Manassas, VA, USA), cultured as recommended and supplemented with 10% FBS. SCp2 cells were grown in Dulbecco's modified Eagle's medium/F-12 (Invitrogen) supplemented with 2% FBS and 5 μg/ml insulin. LM3 and LMM3 were cultured in MEM (Hyclone), supplemented with 5% FBS (Invitrogen). All cell lines were cultured with antibiotic-antimycotic (100 units/ml penicillin G sodium, 100 μg/ml streptomycin sulfate, 250 ng/ml amphotericin B as Fungizone (Invitrogen)) at 37°C in a humidified atmosphere with 5% CO_2 _in air.

### Primary cultures

MMTV(LA)-induced tumor primary cultures (TPCs) were prepared from HITs (that are, estrogen receptor-negative, progesterone receptor-negative (ER^-^PR^-^) neoplasias) (see Table 1). Tumor pieces were washed in MEM with antibiotic-antimycotic and passed through a nylon mesh. Sediment was resuspended in 20 ml of MEM containing 1% FBS and allowed to precipitate for 20 minutes. The liquid phase of the suspension was removed and cells were plated into 60 mm tissue culture dishes or six-well plates. Cells were cultured in MEM containing 1% FBS and 10 ng/ml epidermal growth factor(Sigma Aldrich, Saint Louis, MO, USA); when the cells had grown to near confluence (3 to 4 days) they were rinsed with PBS and incubated in MEM containing 1% FBS for 24 to 36 hours before experimental treatments. Culture dishes and plates were precoated by incubating them for 1 hour at room temperature (18–22°C) with 50 μg/ml collagen in 0.02 M acetic acid (rat tail collagen, type 1; Becton Dickinson Labware, Franklin Lakes, NJ). The remaining solution was carefully aspirated and then rinsed with PBS. For the preparation of conditioned medium (CM), established primary cultures were grown in serum-free MEM for 15 hours and then in MEM containing 1% FBS for 2 days before the supernatant was collected. CM was then mixed with fresh medium to final proportions of 30%, 50% and 80%.

### Biological and chemical reagents

Recombinant murine LIF (Sigma Aldrich) concentration is indicated in each experiment. Recombinant murine IL-6 (Sigma Aldrich) was used at 80 ng/ml. For neutralization of LIF, 1 ml of CM was incubated with 0.8 μg of anti-mLIF neutralizing antibody (R&D Systems, Minneapolis, MN, USA) at room temperature for 1 hour before cell treatment [27]. To inhibit extracellular signal-regulated kinase (ERK)1/2 activbation serum-starved HC11 cells were pretreated for 1 hour with 30 μM PD98059 (Calbiochem, San Diego California) or with vehicle (1% v/v dimethyl sulfoxide) and then treated with LIF for 5 minutes (for Western blot analysis) or 72 hours (for cell viability assays). Treatment with Src inhibitor (PP2; Calbiochem) was performed as described previously [28]. In brief, HC11 were starved for 1 hour and preincubated with 30 μM PP2 for 15 minutes before treatment with LIF for 5 minutes. In order to inhibit Stat3 activation, cell cultures were pretreated with 1 mM Stat3-specific inhibitory peptide (Stat3ip; Calbiochem) 1 hour before stimulation with LIF for the indicated periods.

### Morphological and immunohistochemical studies

Tumors and normal mammary glands were fixed in 10% buffered formalin and embedded in paraffin by using standard procedures [17]. In brief, after paraffin sections had been dewaxed, they were rehydrated and either stained with hematoxylin and eosin or used for immunohistochemical studies. LIF immunohistochemistry was performed as described [17] with a polyclonal mouse LIF antibody (SC-1336). Stat3 immunohistochemistry was conducted with a polyclonal rabbit anti-Stat3 antibody (SC-482) (Santa Cruz Biotechnology, Inc., Santa Cruz, CA, USA). Detections were performed with the Vectostain Elite ABC immunoperoxidase system (Vector Laboratories, Burlingame, CA, USA) in accordance with the manufacturer's instructions with diaminobenzidine (Dako, Carpinteria, CA, USA) as chromogen. LIF and Stat3 immunostaining were qualitatively evaluated by: (1) the presence or absence of staining; (2) the type of structure with positive staining and (3) the pattern and/or cellular localization of staining. Negative controls were performed by replacing the primary antibody with normal rabbit serum.

### Immunofluorescence

HC11 were cultured on Lab-tek chamber slides (NUNC, Rochester, NY, USA) for 48 hours, then preincubated with Stat3 inhibitor peptide for 1 hour and treated with LIF (50 ng/ml) for 30 minutes. After that, cells were fixed in 4% paraformaldehyde for 25 minutes at room temperature, washed with PBS and preincubated at room temperature for 5 minutes with PBS-based blocking buffer containing 0.1% SDS and 3% bovine serum albumin. After being rinsed with PBS, the cells were incubated with a 1:100 dilution of rabbit polyclonal anti-Stat3 antibody (SC-482, Santa Cruz Biotechnology) in the same blocking buffer. After being washed with PBS, cells were incubated for 1 hour with a 1:500 dilution of Cy3-conjugated affiniPure donkey anti-rabbit IgG (1.5 mg/ml; Jackson Immunoresearch Laboratories, West Grove, PA, USA).

Cells were mounted and observed under an Olympus Fluoview FV300 Confocal Laser Scanning Biological Microscope. Images were analyzed by using Adobe Photoshop (Adobe Systems, Inc., New York, NY, USA).

### Protein extraction

Total proteins were extracted from frozen mammary glands, frozen tumor tissue or cell lines in RIPA protein extraction buffer (50 mM Tris-HCl pH 7.4, 150 mM NaCl, 1% Triton X-100, 0.25% sodium deoxycholate, 1 mM EDTA) supplemented with protease (protease inhibitor cocktail set I; Calbiochem) and phosphatase inhibitors (1 mM NaF and 1 mM Na_2_VO_4_). Samples were homogenized and further disrupted by passage through a 21-gauge needle (8 to 10 times). They were subsequently incubated on ice for 30 minutes and centrifuged at 9,500 *g *for 20 minutes at 4°C. Supernatants were transferred to a fresh tube and the protein concentration was determined by the Bradford method [29]. Cleared lysates were combined with SDS sample buffer (50 mM Tris-HCl pH 6.8, 2% SDS, 0.1% bromophenol blue, 10% glycerol, 100 mM dithiothreitol), boiled for 8 minutes and resolved by SDS-PAGE.

### Immunoprecipitation

Protein extracts (1.5 mg) from mouse tumors were incubated with 7 μl of anti-Stat3 (C-20; Santa Cruz Biotechnology) at 4°C overnight, with horizontal rotation. Protein A/G-Sepharose beads (Santa Cruz Biotechnology) were added and incubation continued for a further 2 hours at room temperature. Samples were then washed three times with PBS and resuspended in 10 μl of the previously described sample buffer.

### Western blot analysis

Proteins were run on 10% SDS-polyacrylamide gels (80 μg per lane), blotted to poly(vinylidene difluoride) membranes (Bio-Rad) and incubated with blocking solution (5% dry skimmed milk dissolved in TBS-T, 50 mM Tris-HCl pH: 8.8, 150 mM NaCl and 0.1%Tween) for 1 hour. A set of prestained molecular mass standards was run in each gel. Membranes were incubated overnight at 4°C with the appropriate dilution of the following primary antibodies: a rabbit polyclonal anti-Stat3 antibody (C-20, SC-482), a mouse monoclonal anti-tyrosine-phosphorylated (pY) Stat3 (SC-8059), a rabbit polyclonal anti-ERK (SC-154) and a mouse monoclonal anti-pY-ERK (SC-7383). All antibodies were purchased from Santa Cruz Biotechnology. Membranes were washed with TBS-T before incubation with horseradish peroxidase-conjugated anti-mouse or anti-rabbit secondary antibodies (Santa Cruz Biotechnology). Immunoreactive protein bands were detected by enhanced chemiluminescence (ECL+Plus System; Amersham Biosciences).

### RNA analysis

Mammary gland and mammary tumor RNA was obtained using the SV Total RNA Isolation System (Promega, Madison, WI, USA) in accordance with the manufacturer's instructions. RNA from cell lines and primary cultures was obtained with Trizol (Invitrogen). For Northern blot analysis, poly(A) RNA was obtained and processed as described previously [17]. For RT-PCR analysis, cDNA was generated from 2 μg of total RNA using Moloney murine leukemia virus reverse transcriptase (Promega), 10 μl of reverse transcription buffer, oligodeoxythymidylic acid primer, 25 mM deoxynucleoside triphosphates mix and RNase inhibitor (Promega) in a final reaction volume of 20 μl (60 minutes at 40°C followed by 5 minutes at 90°C). The primers and amplification protocol used in detecting LIF, LIF-R and actin expression have been reported previously [17]. For gp130, the sense and antisense primers used were 5'-TCGGAGGAGCGGCCAGAAGAC-3' and 5'-ATCAGCCCCCGTGCCAAGAGC-3', respectively. For CCAAT-enhancer-binding protein (C/EBP)δ, the sense and antisense primers used were 5'-ACCCGCGGCCTTCTACGA-3' and 5'-CGCCCCTTTTCTCGGACTGT-3', respectively. Products were subjected to electrophoresis in 2% agarose gels. For detection of LIF-M and LIF-D expression, the sense primer sequence for LIF-M was 5'-TGGAAAGCTGTGATTGGCGCGAGA-3' and that for LIF-D was 5'-TGGAGTCCAGCCCATAATGAAGGT-3' ; in both cases a primer from exon 3 was used (5'-TGGAGTCCAGCCCATAATGAAGGT-3') and the PCR was performed with 35 amplification cycles. The products were subjected to electrophoresis on 2% agarose gel.

Real-time PCR data were acquired and analyzed with an Opticon Monitor System (MJ Research, Bio-RAD, Hercules CA, USA) and each amplification mixture was performed in 3.5 mM MgCl_2_, 4 μM forward primer, 4 μM reverse primer, 1:30,000 SYBR Green (Invitrogen) with the previously described RT-PCR kit and protocols [17] but using 35 amplification cycles. All samples were analyzed for actin expression in parallel in the same run. For each sample, the amplification plot and the corresponding dissociation curves were examined. The specificity of the amplified product was monitored by examining the melting curve and the melting peak of the product. The absence of nonspecific amplification was confirmed for each gene by analyzing the PCR amplification products by agarose gel electrophoresis. To estimate mRNA expression, calibration curves were made. Experiments were always run in duplicate and repeated at least twice.

### Cell viability assays

Cell viability was evaluated by staining with crystal violet as described previously [30]. In brief, cells were treated as indicated and fixed with 1.1% glutaraldehyde at the end of each experiment. After being washed with deionized water and dried in air, plates were stained with a 0.1% crystal violet solution. The bound dye was solubilized with 10% acetic acid and quantified at 590 nm in a Benchmark microplates reader (Bio-Rad Laboratories, Hercules, CA, USA).

## Results

### Expression of leukemia inhibitory factor (LIF) and LIF receptor in mouse mammary tumor cells

**Figure 1:**
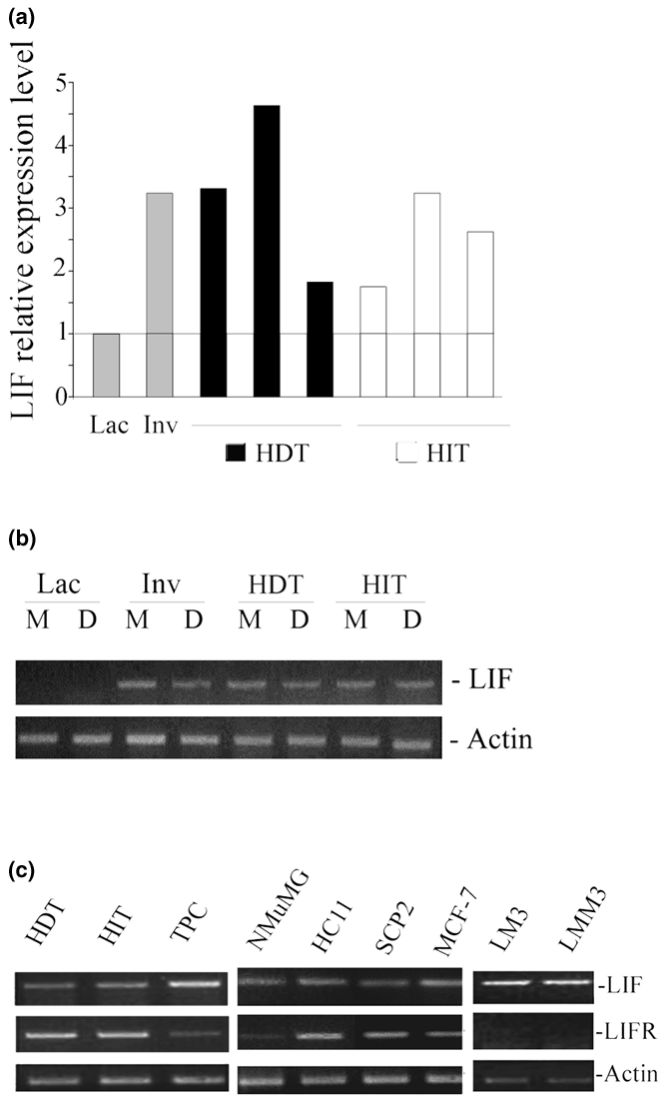
Expression of LIF and LIF receptor mRNA in mammary tumor cells. **(a) **Real-time PCR showing relative expression levels of leukemia inhibitory factor (LIF) in hormone-dependent (HDT) and hormone-independent (HIT) mouse mammary tumors compared with lactating (Lac) and 48-hour involuting (Inv) normal mammary glands. All samples were analyzed and normalized to actin expression in parallel in the same real-time PCR assay. **(b) **Ethidium bromide stained gel showing RT-PCR analysis of two different LIF splicing variants: LIF-M and LIF-D. **(c) **Ethidium bromide stained gel showing LIF and LIF-R expression by RT-PCR in HDT, HIT and in culture growing mammary cell lines: NMuMG, HC11, SCp2 (epithelial non-tumorigenic), MCF-7, LM3, LMM3 (tumorigenic mammary cell lines) and mammary tumor primary cultures (TPC).

First, LIF expression was analyzed in MMTV(LA) induced HDTs and HITs (see Table 1) [24,25,31]. When the LIF mRNA content from different HDTs (*n *= 3) and HITs (*n *= 3) was compared with lactating and involuting normal mammary glands, we found (by quantitative RT-PCR) that this cytokine was expressed in all these tumors, although its level varied between them independently of its dependence on hormone (Fig. 1a). These results were confirmed by Northern blot analysis (data not shown). Because it has been demonstrated that there are two alternatively spliced LIF transcripts that originate two differentially localized LIF proteins, a secreted 'free' form (LIF-D) and an extracellular matrix-associated one (LIF-M) [32], their presence was analyzed by RT-PCR with specific sets of primers. Our results show that both the LIF-D and LIF-M transcripts are present in mammary tumors and involuting glands (Fig. 1b). To determine the relevance of autocrine LIF in mammary cells, the expression of LIF and LIF-R was determined by RT-PCR in HDTs, HITs, their primary cultures, and mouse (non-tumorigenic and tumorigenic) and human (tumorigenic) mammary cell lines (NmuMG, HC11, SCp2, LM3, LMM3 and MCF-7; Table 1). We found that all those cells express LIF, although tumor cells tend to show higher levels than non-tumorigenic ones. Interestingly, LIF-R was also present in all cell lines tested, with the exception of LM3 and LMM3 (Fig. 1c).

### LIF expression and Stat3 localization analysis; histological studies

**Figure 2:**
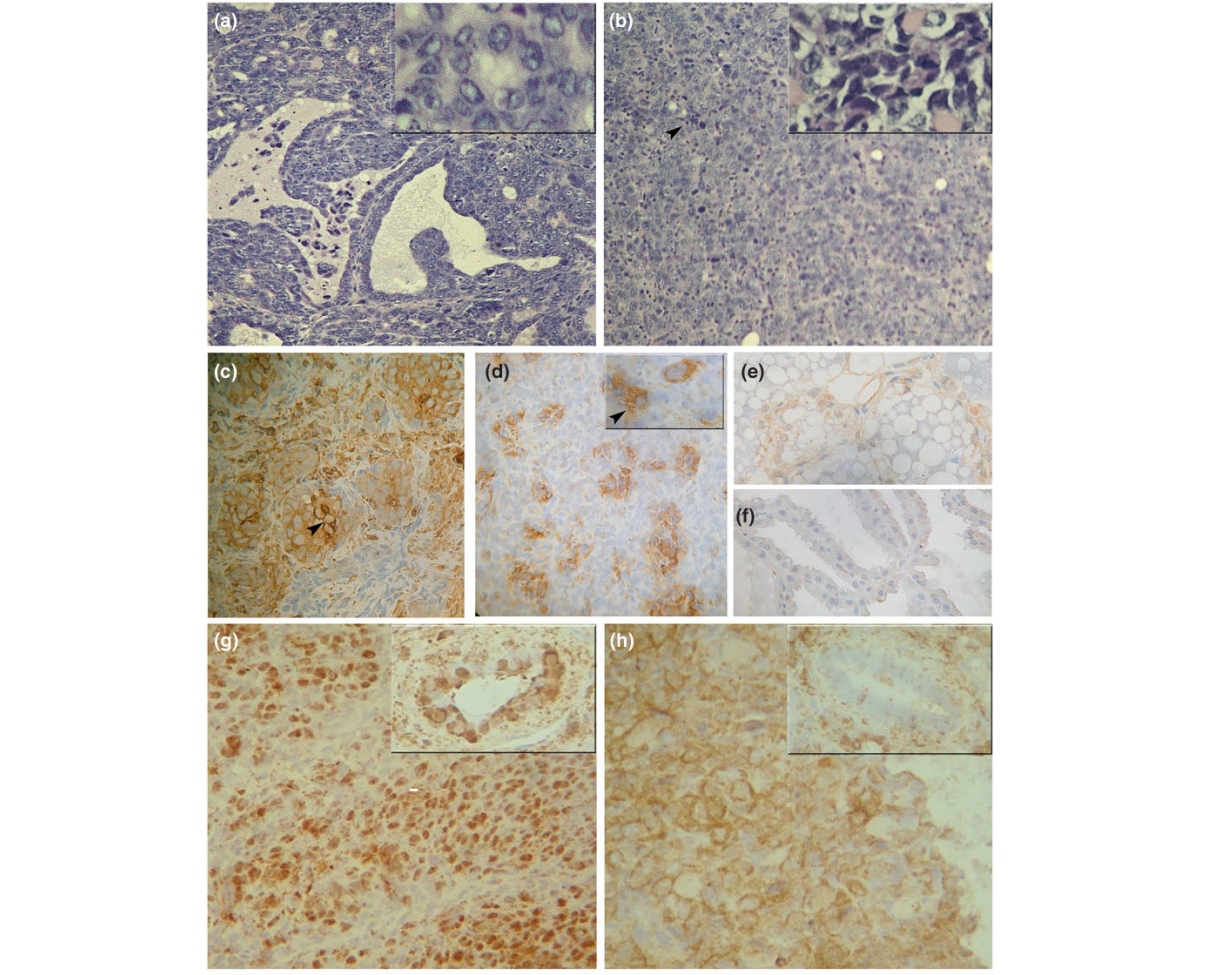
Morphological and immunohistochemical analysis of MMTV(LA)-induced HIT and LM3 mammary tumors growing *in vivo*. **(a, b) **Hematoxylin/eosin-stained tumors. **(a) **Moderately differentiated hormone-independent tumors (HIT). Papillary formations into lumen of cystic ducts and less differentiated zones with small glandular structures. Original magnification ×100; inset ×400. **(b) **LM3 poorly differentiated adenocarcinoma with abundant infiltrated vascular stroma. Areas with sarcomatous appearance and apoptotic images (black arrowheads). Original magnification in (a, b) ×100; inset ×400. **(c-f) **Leukemia inhibitory factor (LIF) immunohistochemistry. **(c) **HIT: heterogenous and regular distribution mainly in poorly differentiated areas with cytoplasmic and nucleic positive staining; intense cytoplasmic staining increasing peripherally indicates a secretory pattern (arrowhead). **(d) **LM3 tumor: patchy LIF staining. Inset: positive cytoplasmic staining; the ill-defined cell membrane pattern with granular cytoplasmic staining indicates LIF secretion (arrowhead). **(e) **Involuting (48-hour) and **(f) **lactating mammary glands were used as positive and negative controls, respectively, for LIF staining. Original magnification in (c-f) 250×; inset: 400× **(g, h) **Stat3 (signal transduction and activators of transcription 3) immunohistochemistry. **(g) **HIT: stromal and predominantly epithelial positive nuclear staining in solid areas. Inset: stained nuclei were also seen in glandular regions. **(h) **LM3 tumor: intense cytoplasmic positive staining in stromal and epithelial cells. The inset shows a negative gland surrounded by positive stromal cells. Original magnification in (g, h) 250×; inset: 400×

Morphologically, MMTV(LA)-induced tumors are classical adenocarcinomas with various grades of differentiation. Figure 2a shows an example of a moderately differentiated HIT with many cystic papillary areas. Although these tumors also show poorly differentiated solid regions, round epithelial cells forming small glandular structures could be observed in these areas (Fig. 2a, inset). In contrast, LM3 tumors are poorly differentiated adenocarcinomas with large tumor cells and hyperchromatic nuclei. They also show an abundant vascular stroma that contains many fibroblasts, neutrophils, lymphocytes, plasma cells, and occasionally mast cells (Fig. 2b). Apoptotic images and extensive hemorrhagic necrosis are also seen. In addition, because of the fusiform feature and swirled disposition of some cells, there are areas with a sarcomatous appearance (Fig. 2b, inset).

LIF expression has been tested by immunohistochemistry in HITs and in LM3 tumors (Table 1). In both cases, LIF staining was predominantly epithelial, although some positive stromal cells could be seen (Fig. 2c, d). The expression of LIF in involuting and lactating mammary glands is shown as a positive and a negative control, respectively (Fig. 2e, f).

**Figure 3:**
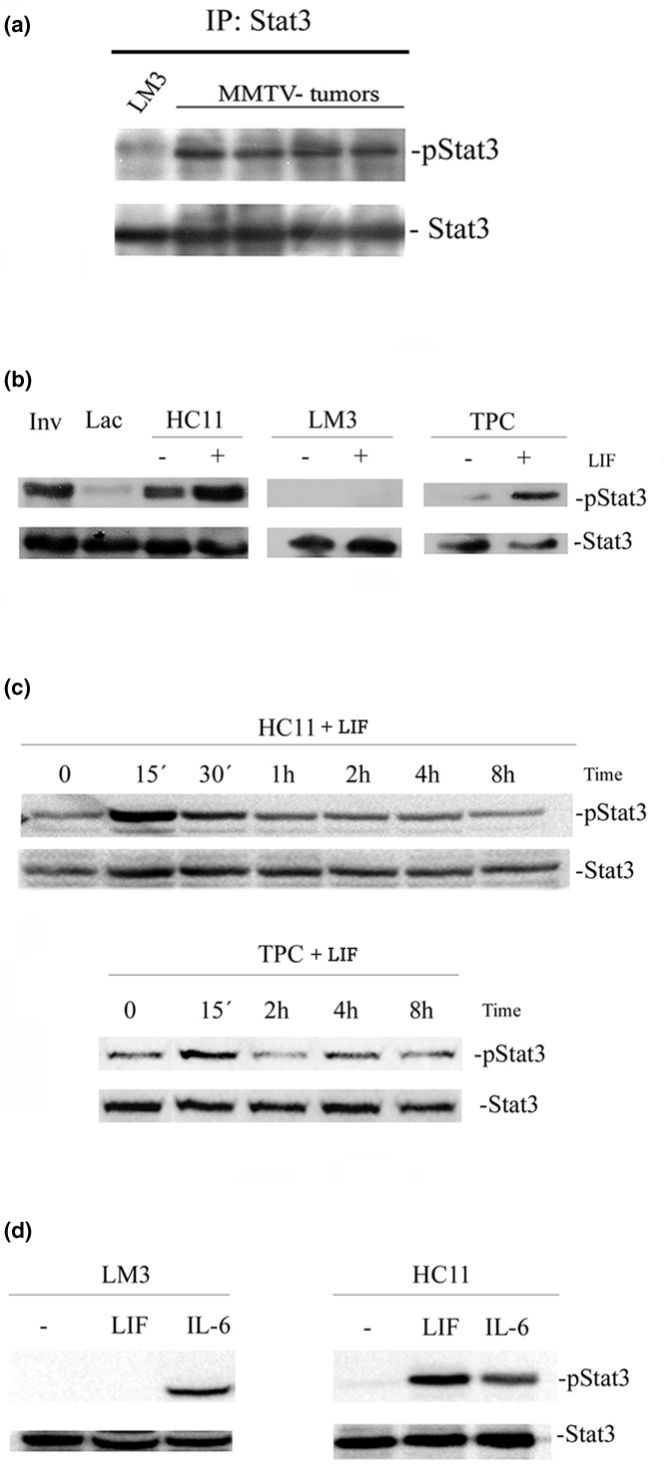
Western blot analysis of phospho-Stat3 (pStat3) and Stat3. **(a) **Tumors growing *in vivo*: LM3 (lane 1) and different HIT transplants (lanes 2 to 5). **(b-d) **Cells growing in culture. **(b) **HC11, LM3 and tumor primary culture (TPC) cells treated with 80 ng/ml leukemia inhibitory factor (LIF) for 15 minutes; mammary glands at 48 hours of involution (Inv) and at fifth day of lactation (Lac) were used as positive and negative controls, respectively. **(c) **Time course of tyrosine phosphorylation of Stat3 (signal transduction and activators of transcription 3) in HC11 cells (upper panel) and TPC cells (lower panel) treated with 80 ng/ml LIF. **(d) **Tyrosine phosphorylation of Stat3 in HC11 and LM3 cells treated with LIF and IL-6 (both at 80 ng/ml) for 15 minutes.

To determine the level of Stat3 activation in HITs and LM3 tumors, its intracellular localization has been determined by immunohistochemical analysis. Whereas in HITs the images show positive staining in epithelial and stromal nuclei (Fig. 2g); in LM3 tumors Stat3 staining was detected mostly in the cytoplasm of epithelial cells (Fig. 2h), which indicates a lack of Stat3 activation in these tumors. This observation was confirmed by Western blot analysis: all the analyzed HITs showed much higher levels of pY-Stat3 than LM3 tumors (Fig. 3a). These results suggest that the lack of LIF-R expression results in a much lower activation of Stat3 in the LM3 tumors.

### Tyrosine phosphorylation of Stat3 in culture

For further analysis of the hypothesis that LIF-mediated signaling would be a determinant for Stat3 activation in mouse mammary tumors, the capacity of LIF to induce tyrosine phosphorylation of Stat3 was analyzed in cultured cells. Our results show that LIF was able to induce transient Stat3 activation in HC11 and TPC cells, achieving the highest level of tyrosine phosphorylation after 15 minutes. However, no pY-Stat3 was observed in LIF-treated LM3 cells (Fig. 3b, c).

To determine the integrity of the gp130/JAK/Stat3 signaling pathway in LM3 cells, gp130 expression and the capacity of another LIF-family cytokine to induce Stat3 phosphorylation was evaluated. We found similar levels of gp130 mRNA in all cells tested (HC11, TPC and LM3; data not shown). In addition, IL-6-treated LM3 cells showed a significant level of pY-Stat3 (Fig. 3d). This suggests that the lack of Stat3 activation in LIF-treated LM3 cells was due to a deficiency in LIF-R expression and not to the impairment of another component of the gp130/JAK/Stat3 signaling cascade.

**Figure 4:**
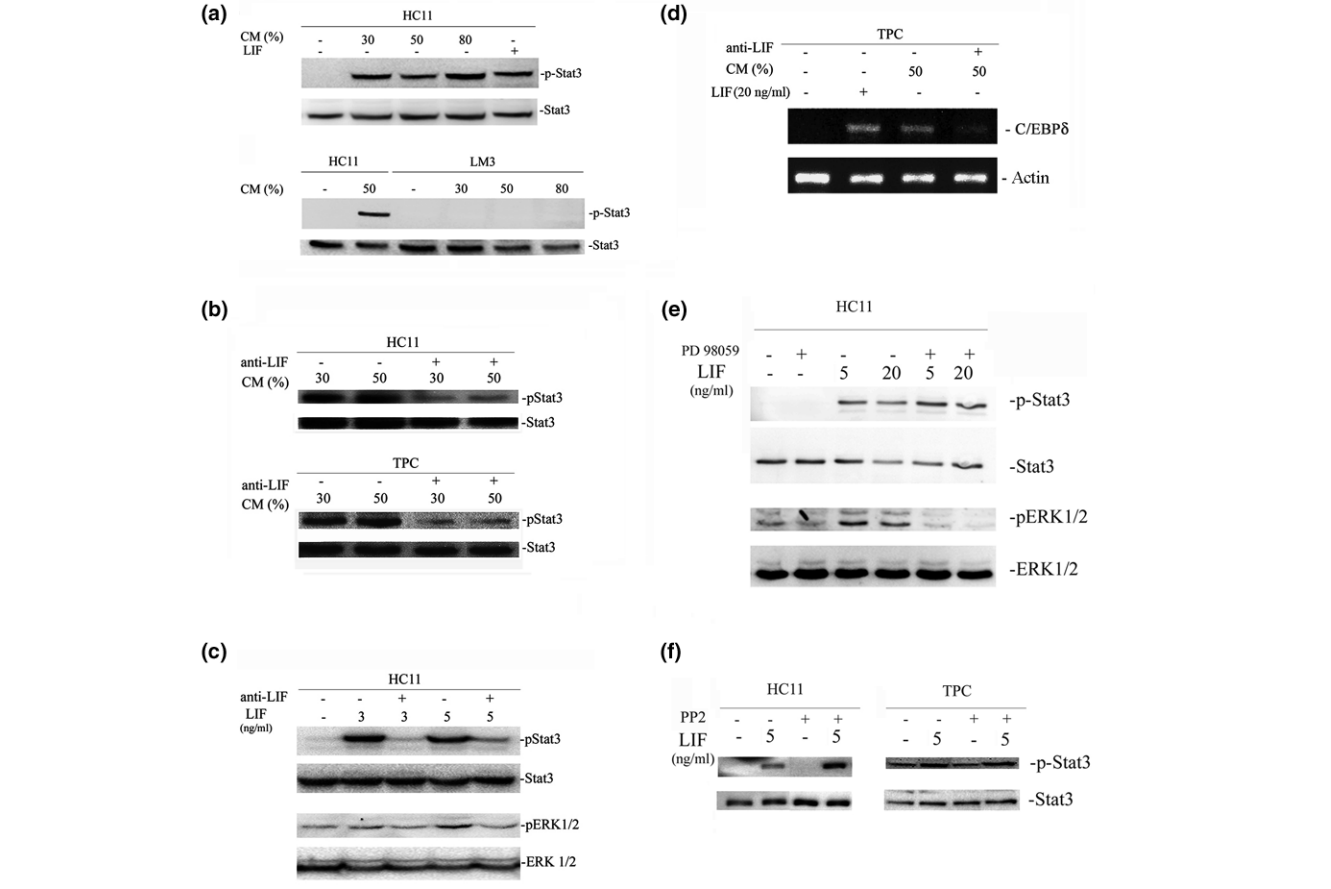
Effect of tumor primary culture (TPC) conditioned medium (CM) and leukemia inhibitory factor (LIF)-neutralizing antibody. **(a) **Stat3 (signal transduction and activators of transcription 3) and phospho-Stat3 (p-Stat3) in HC11 and LM3 cells treated with increasing concentrations of CM (30%, 50% and 80%). **(b) **Stat3 and p-Stat3 levels in HC11 and TPC cells treated with CM that had been preincubated with or without LIF-neutralizing antibody. **(c) **Phosphorylation levels of Stat3 and extracellular signal-regulated kinase (ERK)1/2 in HC11 cells treated with LIF (3 and 5 ng/ml) preincubated with or without LIF-neutralizing antibody. **(d) **Expression of CCAAT-enhancer-binding protein (C/EBP)δ in TPC cells treated with CM with or without neutralizing antibody. **(e) **Phosphorylation of Stat3 and ERK1/2 in HC11 cells treated with LIF with or without the MAP kinase/ERK kinase inhibitor PD98059. **(f) **p-Stat3 and Stat3 in HC11 and TPC cells treated with LIF with or without the Src-specific inhibitor PP2. Experiments were repeated at least three times with similar results. No effect was observed on phosphorylation levels of either Stat3 or ERK1/2 when HC11 cells were treated with the PD98059 vehicle, dimethyl sulfoxide (data not shown).

We next investigated the capacity of TPC CM to induce Stat3 phosphorylation in mammary cells. Our results show that CM induced Stat3 phosphorylation in HC11 cells (Fig. 4a, upper panel). Interestingly, this treatment was unable to induce Stat3 activation in LM3 cells (Fig. 4a, lower panel). A LIF blocking antibody was then used to determine whether this cytokine was responsible for the CM-induced Stat3 activation. Our results show a clear inhibition of this activity in HC11 and TPC cells (Fig. 4b). Notably, this antibody was unable to completely block the capacity of LIF (3 to 5 ng/ml) to induce Stat3 phosphorylation in HC11 cells (Fig. 4c). The remaining Stat3 activation observed in cells treated with CM plus LIF-blocking antibody could therefore still have been due to residual LIF activity in the presence of this antibody. These results indicate that locally produced LIF exerts a major role on Stat3 tyrosine phosphorylation in mammary tumors.

To determine whether Stat3 tyrosine phosphorylation induced by CM resulted in transcriptional activation of this factor, we assessed the expression of a known transcriptional target of Stat3, namely C/EBPδ [33]. Our results show that LIF as well as CM induces C/EBPδ transcription in mammary tumor cells and that CM-dependent C/EBPδ induction was inhibited by pretreatment with LIF-blocking antibody (Fig. 4d).

It has been reported that the IL-6 cytokine family is able to induce Stat3 activation through the gp130 receptor by using an 'unconventional' signaling route that involves ERK1/2 phosphorylation [34]. The ability of LIF to induce this mitogen-activated protein kinase (MAPK) activation was then evaluated in HC11 cells. LIF (5 ng/ml) induced a detectable activation of ERK1/2 that disappeared in the presence of LIF-blocking antibody (Fig. 4c). However, the use of a MAPK/ERK kinase (MEK)-specific inhibitor (PD98059) completely blocked LIF-induced ERK1/2 activation but did not affect the induction of Stat3 tyrosine phosphorylation (Fig. 4e). These results indicate that the ERK1/2 activation achieved with 5 to 20 ng/ml LIF does not exert a major effect on Stat3 activation in HC11 cells. In addition, PP2, a selective inhibitor of Src family of protein tyrosine kinases, had no effect on LIF-induced Stat3 tyrosine phosphorylation in mammary cells (Fig. 4f), suggesting that this effect would not depend on Src activation.

**Figure 5:**
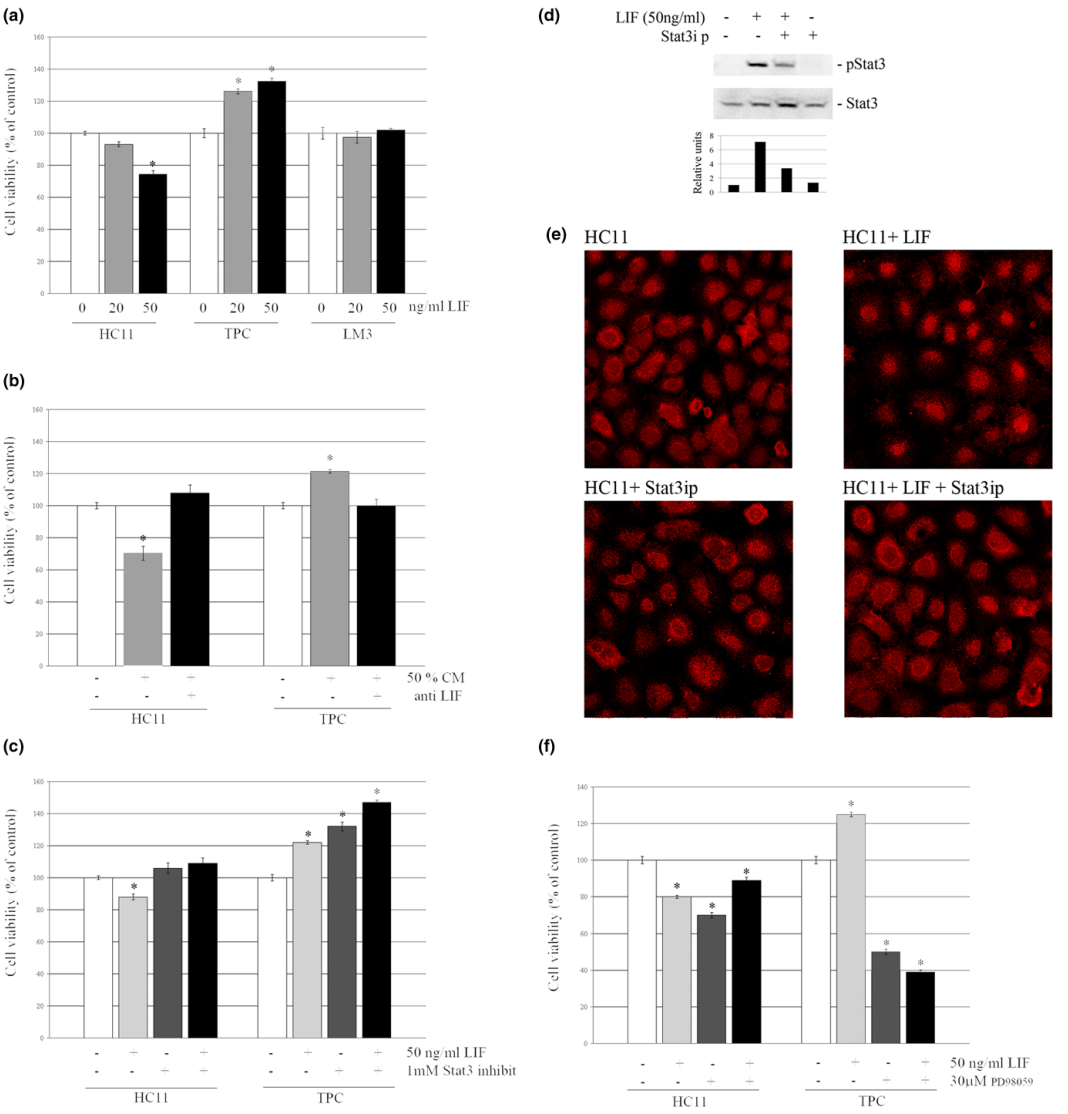
Effect of LIF and CM on cell viability after 72 hours of treatment. Viability was assessed by crystal violet assays. **(a) **HC11, tumor primary culture (TPC) and LM3 cells were treated with leukemia inhibitory factor (LIF; 20 and 50 ng/ml). **(b) **HC11 and TPC cells were treated with 50% conditioned medium (CM) preincubated with or without LIF-neutralizing antibody. **(c) **HC11 and TPC cells were treated with 50 ng/ml LIF in the presence or absence of a Stat3 (signal transduction and activators of transcription 3) inhibitor peptide. **(d, e) **Effect of Stat3-specific inhibitory peptide (Stat3ip) on Stat3 phosphorylation levels (d) and nuclear translocation (e) induced by LIF. 400× **(f) **HC11 and TPC cells were treated with 50 ng/ml LIF in the presence or absence of PD98059. Data are percentages of internal control for each cell type (time 0) and are expressed as means ± SEM for four replicates. Experiments were repeated at least three times with similar results. Asterisk denotes statistical difference (*P *< 0.05) in a two-tailed Student's *t *test.

To analyze the biological activity of LIF on mouse mammary tumor and non-tumor cells, we evaluated the effect of this cytokine on the survival of HC11, TPC and LM3 cells. We have found that 72 hours of LIF treatment induced a dose-dependent inhibition of HC11 cell survival, whereas it also caused a dose-dependent increase in the number of viable primary tumor cells. As expected, no effect was observed in LIF-treated LM3 cells (Fig. 5a). Similarly, CM induced opposite effects on the viability of HC11 and TPC cells; these were prevented by pretreatment with a LIF-blocking antibody (Fig. 5b).

Then, to determine whether Stat3 and/or ERK1/2 activation were involved in the effect of LIF on cell survival, HC11 and TPC cells were treated with this cytokine for 72 hours in the presence or absence of Stat3ip or PD98059. As expected, the inhibitory effect of LIF on the survival of HC11 cells was blocked by Stat3ip. Interestingly, this treatment did not inhibit the stimulatory activity of LIF on TPC cell survival; instead, it significantly enhanced it (Fig. 5c). In contrast, blocking ERK1/2 activation had a marked inhibitory effect on TPC cell survival, whereas the same assay produced a much milder response in HC11 cells (Fig. 5f). As has previously been reported by others, we confirmed the inhibitory capacity of Stat3ip (1 mM) by analyzing its ability to interfere with LIF-induced Stat3 phosphorylation (Fig. 5d) [35] and nuclear translocation (Fig. 5e) [36]. Therefore, the different biological activity displayed by LIF on normal cells and tumor cells might be due to the altered biological response that tumor cells develop to the activation of certain signaling pathways rather than to a differential effect of this cytokine on its intracellular mediators.

## Discussion

Activation of Stat3 has been detected in many human neoplasias [37,38], and it has been shown that IL-6-type cytokines induce Stat3 phosphorylation in various human and rodent cell lines [21,23]. In addition, it has been demonstrated that these cytokines, including LIF, are expressed in breast cancer cells and in other tumor types [4,39]. Interestingly, in certain myeloma and prostate cancer cell lines, IL-6 has been identified as the main cytokine responsible for Stat3 activation induction [40,41]. In addition, a very recent report suggests a similar role for this cytokine in breast cancer cells [42]. In mouse mammary glands during post-lactational involution, both induction of IL-6 and LIF expression and Stat3 activation have been demonstrated [17,18,34]. Interestingly, in this context, Stat3 activation seems to be more dependent on LIF than on IL-6 status [43]. In mammary tumors, to our knowledge, no report has yet been made linking LIF expression to Stat3 activation.

It has been reported that LIF and LIF-R expression in breast tumors is associated with favorable biological features such as diploidy and low S-phase fraction. In addition, in those tumors LIF-R expression was correlated with the presence of ER [3]. On the basis of these data, those authors postulated that tumors expressing LIF/LIF-R would represent a phenotype that is closer to 'normal' and would therefore be less aggressive. Interestingly, although it has been shown that Stat3 expression and activation is commonly found in breast cancer cells [15], and this transcription factor activation resulted in the malignant transformation of fibroblasts [43] and the proliferation of mammary tumor cells [4,6,44,45], there is also evidence that Stat3 activation in human breast cancer is associated with a better prognosis [46].

Our results in mouse mammary tumors also show an association between LIF-R expression and Stat3 activation with a less aggressive phenotype. LIF-R^+ ^MMTV(LA)-induced tumors appear in mid-pregnancy, when serum progesterone and estrogen levels are high, and continue to grow until delivery. After this, they soon regress and reappear in subsequent gestations, suggesting that progesterone and/or estrogen have a main role in their development. In addition, these tumors show high expression of estrogen and progesterone receptors (ER^+^PR^+^). Eventually, in the same mouse or after successive passages, these neoplasias progress to become autonomous: they lose hormone receptor expression and grow independently of the female's hormonal status [24]. However, in spite of this drastic change in hormone dependence, MMTV(LA) HITs are not very aggressive and show a variety of histological patterns, from well-differentiated to very poorly differentiated architecture [24]. They also remain LIF responsive and show Stat3 activation. In contrast, the LM3 cell line, which derives from a spontaneous BALB/c mammary adenocarcinoma, gives rise to ER^- ^PR^- ^poorly differentiated highly invasive (100% incidence of lung metastasis) tumors [47]. Here we have shown that these tumors do not express LIF-R and show low levels of Stat3 activation. Our results from mouse mammary tumors are therefore in good agreement with data from human breast cancer samples. This suggests that, in certain cases, these experimental models can be better tools than breast cancer cell lines for reproducing particular aspects of human malignancies.

In transformed cells, there are no known naturally occurring mutations in Stat3 that lead to its constitutive activation. Alternatively, it has been proposed that Stat3 activation in tumors and in oncogene-transformed cells would be dependent on growth factor tyrosine kinase receptor activation or deregulation of JAK kinase's activity [48]. The results shown here demonstrate that in well-differentiated mouse mammary tumors the constitutive activation of Stat3 would be mostly dependent on overexpression of LIF. The phosphorylation regulatory pathways of this transcription factor might therefore not be altered in these cancer cells. In addition, it has been shown that blocking ERK1/2 phosphorylation resulted in inhibition of Stat3 activation in Jak2-null cells, whereas no effect on pY-Stat3 has been observed in wild-type cells [43]. Similarly, in HC11 cells we have not found a clear effect on Stat3 tyrosine phosphorylation when ERK1/2 activation was blocked. These results suggest that this MAPK could have a relevant role in mammary Stat3 activation only when the gp130/Jak2 pathway has been impaired in some way.

Our results from the crystal violet assays indicate that treatment with LIF can produce different biological responses in non-tumorigenic and tumorigenic cells, namely inhibiting and inducing cell survival, respectively. However, and in spite of this cytokine's being the principal one responsible for Stat3 phosphorylation, in both cases blocking Stat3 activation increased cell survival. It has been shown that expression of the phosphoinositide 3-kinase regulatory subunits p55α and p50α is directly induced by Stat3 during mammary gland involution [18]. These proteins are involved in the downregulation of phosphoinositide 3-kinase signalling and Akt/protein kinase B activity, and abrogation of this survival pathway is essential for the induction of apoptosis in mammary epithelial cells [18]. Our interpretation for the results shown here is therefore that in certain mammary tumor cells this apoptosis-inducing pathway is still functioning. However, these cancer cells are not fully responsive to the strategies for controlling cell survival because they are very sensitive to the activation of proliferative signaling pathways. For example, our results show clearly how much more susceptible tumor cells are to inhibition of MEK activity. We therefore believe that in both normal and neoplastic mammary cells LIF is able to induce both the survival and apoptotic pathways, the balance of which can lead to completely different outcomes in these cell types.

Stat3 biological activity depends on multiple factors, many still unknown. For example, in melanoma cells, IL-6/Stat3 function is modulated by the stage of tumor progression [49]. The results shown here suggest that in well-to-moderately differentiated mammary tumor cells, LIF-induced Stat3 activation preserves the pro-apoptotic role of this factor in non-tumorigenic mammary cells. This activity might be altered in more aggressive or less differentiated tumors by different causes that need to be analyzed in future experiments. However, our results imply that in the development of therapeutic strategies for blocking Stat3 in breast cancer cells, the strong dependence on the cellular context that this factor activity displays should be taken into account.

## Conclusion

The results presented here show that LIF is overexpressed in MMTV-induced mammary carcinomas, in which, as a paracrine/autocrine factor, it is the main one responsible for Stat3 activation. In well-differentiated mammary cancer cells, constitutive activation of Stat3 would therefore depend on LIF and LIF-R expression, as occurs in normal mammary epithelium. However, in these cancer cells, LIF induces cell survival through signaling pathways that would not involve Stat3 activation.

## Abbreviations

C/EBP = CCAAT-enhancer-binding protein; CM = conditioned medium; ER = estrogen receptor; ERK = extracellular signal-regulated kinase; FBS = fetal bovine serum; HDT = hormone-dependent tumor; HIT = hormone-independent tumor; IL = interleukin; JAK = Janus kinase; LIF = leukemia inhibitory factor; LIF-D = secreted 'free' form of LIF; LIF-M = extracellular matrix-associated form of LIF; LIF-R = leukemia inhibitory factor receptor; MAPK = mitogen-activated protein kinase; MEM = minimal essential medium; MEK = MAPK/ERK kinase; MMTV = mouse mammary tumor virus; PBS = phosphate-buffered saline; PR = progesterone receptor; pY = tyrosine-phosphorylated; RT-PCR = reverse transcriptase-mediated polymerase chain reaction; Stat = signal transduction and activators of transcription; Stat3ip = Stat3-specific inhibitory peptide; TPC = tumor primary culture.

## Competing interests

The authors declare that they have no competing interests.

## Authors' contributions

AQ conducted the experiments in culture, the immunoassays and the western blot analysis and assisted in writing the manuscript. CSL performed the RT-PCR and helped in drafting the manuscript. LR helped to perform the crystal violet assays and the RT-PCR of EBP. RPM helped design and perform the morphological and immunohistochemical studies. ECK designed and coordinated the experiments and wrote the final manuscript. All authors read and approved the final manuscript.
